# Pipeline esophageal varices: Insights from clinical cases and models

**DOI:** 10.1002/deo2.70054

**Published:** 2025-01-07

**Authors:** Keita Maki, Hiroaki Haga, Kyoko Hoshikawa, Tomohiro Katsumi, Fumiya Suzuki, Fumi Uchiyama, Yoshiyuki Ueno

**Affiliations:** ^1^ Department of Gastroenterology Yamagata University Faculty of Medicine Yamagata Japan

**Keywords:** endoscopic injection sclerotherapy, endoscopic variceal ligation, esophageal varices, giant esophageal varices, pipeline esophageal varices

## Abstract

**Objectives:**

While esophageal varices (EVs) are typically treated endoscopically, other options such as interventional radiology or surgical treatment are considered when endoscopic treatment is challenging. Pipeline EVs are difficult to treat endoscopically due to their large diameter, and currently, no specific treatment guidelines have been established.

**Methods:**

We reviewed cases of pipeline EVs treated at our hospital and analyzed previously reported cases to collect evidence for the formulation of treatment guidelines. Additionally, we created EV simple models to evaluate the safety margin of endoscopic variceal ligation for varices.

**Results:**

Our analysis included 14 cases of pipeline EVs (four cases treated at our hospital from 2013 to 2024 and 10 previously reported cases from 1990 to 2024). Endoscopic treatment alone was insufficient in six cases (42.9%), and five cases (35.7%) required interventional radiology or surgical intervention. Using EV simple models with varying diameters, EVL was inadequate for varices with diameters of 20 mm or larger.

**Conclusions:**

There are few reported cases of pipeline EVs, making it difficult to determine a treatment algorithm. In our study using an EV simple model, it was suggested that endoscopic variceal ligation is effective in blocking blood flow for EVs with a diameter of 15 mm or less. It is important that we understand there are EVs, such as pipeline EVs, for which there are limitations to safely occluding blood flow with endoscopic variceal ligation, and it may be necessary to develop treatment strategies that include methods other than endoscopic therapy.

## INTRODUCTION

Pipeline esophageal varices (EVs) are a specific type of EVs, and portal vein imaging shows that the varices run like a pipeline from the left gastric vein, through the gastric cardia and esophagogastric junction, to the middle and upper esophagus, without passing through the bamboo blind vessels of the lower esophagus. They were named pipeline varices due to this distinct portal vein hemodynamics.^1^ Pipeline EVs are estimated to account for approximately 2%–4% of all EVs.^1^

Pipeline EVs bypass the palisade vessels, leading to elevated pressure and increased blood flow rates.^2^, ^3^ This elevated pressure dilutes the sclerosing agent injected during treatment, preventing it from efficiently damaging the vascular endothelial cells. Consequently, this makes endoscopic injection sclerotherapy (EIS) more challenging.^4^ However, no definitive treatment strategy for pipeline EVs has been established.

In this study, we reviewed cases of pipeline EVs treated at our hospital, along with previously reported cases. Additionally, we developed EV simple models using polyethylene resin to examine the variceal diameter suitable for effective ligation in endoscopic variceal ligation (EVL). According to the available literature, this is the first report to assess the feasibility of achieving sufficient ligation and blood flow occlusion with EVL in pipeline EVs. The results of our research are expected to guide the treatment plan for pipeline EVs.

## METHODS

### Participants

The study included 183 patients with EVs treated at our hospital between 2013 and 2024 (Table 1). Endoscopic examinations were performed to evaluate the location (Ls/Lm/Li), presence or absence of gastric varices (GVs), form (F1/F2/F3), and red color signs, in accordance with the portal hypertension treatment guidelines. Contrast‐enhanced computed tomography (CE‐CT) scans were conducted to assess the maximum diameter of the EVs, type of blood supply (left gastric vein [LGV], posterior gastric vein, or short gastric vein), the maximum diameter of the blood supply, and the presence or absence of palisading vessels, liver cirrhosis, ascites, and hepatocellular carcinoma. When multiple blood supply routes were present, the maximum diameter of the largest route was recorded. The presence or absence of palisading vessels was determined through fluoroscopic imaging during EIS; when fluoroscopic images were unavailable, CT scans were used.

**TABLE 1:** Characteristics of participants.

Characteristics (*n* = 179)	Typical esophageal varices (*n* = 179)	Pipeline esophageal varices (*n* = 4)
Age, median (range)	67 (16–91)	68 (57–70)
Sex, male/female	119/60	3/1
Etiology, *n* (%)		
HBV	6 (3.4)	0 (0)
HCV	38 (21.2)	2 (50)
non‐B, non‐C	5 (2.8)	0 (0)
Alcohol	65 (36.3)	0 (0)
NASH	24 (13.4)	0 (0)
AIH	6 (3.4)	0 (0)
PBC	18 (10)	0 (0)
IPH	3 (1.7)	2 (50)
EPVO	7 (3.9)	0 (0)
Sarcoidosis	1 (0.5)	0 (0)
After liver resection	3 (1.7)	0 (0)
Liver metastases	3 (1.7)	0 (0)
Child‐Pugh grade, *n* (%)		
Grade A	85 (47.5)	4 (100)
Grade B	81 (45.2)	0 (0)
Grade C	13 (7.3)	0 (0)
mALBI grade, *n* (%)		
Grade 1	19 (10.6)	1 (25)
Grade 2a	29 (16.2)	0 (0)
Grade 2b	86 (48)	3 (75)
Grade 3	45 (25.1)	0 (0)
ALBI score, median (range)	−1.800 (−3.126 to −0.336)	−2.066 (−2.990 to −1.936)
Treatment status, *n* (%)		
Elective	120 (67)	3 (75)
Emergency	59 (33)	1 (25)
Treatment procedure, *n* (%)		
EIS	136 (76)	4 (100)
EVL	42 (23.5)	0 (0)
SB‐tube	1 (0.5)	0 (0)
Location, *n* (%)		
Ls	51 (28.5)	2 (50)
Lm	97 (54.2)	2 (50)
Li	31 (17.3)	0 (0)
Form, *n* (%)		
F1	9 (5)	0 (0)
F2	116 (64.8)	0 (0)
F3	54 (30.2)	4 (100)
GV (+/−), color, RC sign, *n* (%)		
GV (+)	51 (28.5)	3 (75)
GV (−)	128 (71.5)	1 (25)
Cb	168 (93.9)	4 (100)
Cw	11 (6.1)	0 (0)
RC0	29 (16.2)	0 (0)
RC1	92 (51.4)	2 (50)
RC2	49 (27.4)	2 (50)
RC3	9 (5)	0 (0)
Size of esophageal varices (mm) (median, range)	6 (3–13)	17.5 (10–25)
Blood supply route, *n* (%)		
LGV	158 (88.2)	4 (100)
PGV	15 (8.4)	0 (0)
SGV	6 (3.4)	0 (0)
Size of blood supply route (mm) (median, range)	5 (2–12)	10 (7–20)
Palisade vein (+), *n* (%)	179 (100)	0 (0)
LC (+/−)	167/12	3/1
Ascites (+/−)	59/120	0/4
HCC (+/−)	37/142	1/3
Blood biochemistry (median, range)		
T‐bil level (mg/dL)	1.3 (0.3–14.8)	1.1 (0.8–1.2)
Alb level (g/dL)	3.2 (1.6–4.6)	3.5 (3.2–4.4)
AST level (U/L)	36 (10–294)	34 (22–41)
ALT level (U/L)	27 (5–177)	33 (19–44)
PLT count (10^3^/µL)	88 (23–439)	74 (53–102)
PT%	81 (32–118)	81 (53–102)
PT‐INR	1.2 (0.9–2.3)	1.2 (1.0–1.6)

Abbreviations: AIH, autoimmune hepatitis; Alb, albumin; ALT, alanine transaminase; AST, aspartate transaminase; Cb, blue varices; Cw, white varices; EIS, endoscopic injection sclerotherapy; EPVO, extrahepatic portal vein obstruction; EVL, endoscopic variceal ligation; F, form: F1, small/straight; F2, enlarged/tortuous; F3, large/coil‐shaped; GV, gastric varices; HBV, hepatitis B virus; HCC, hepatocellular carcinoma; HCV, hepatitis C virus; IPH, idiopathic portal hypertension; LC, liver cirrhosis; LGV, left gastric vein; Li, locus inferior; Lm, locus medialis; Ls, locus superior; mALBI, modified albumin‐bilirubin; NASH, nonalcoholic steatohepatitis; PBC, primary biliary cholangitis; PGV, posterior gastric vein; PLT, platelet count; PT, prothrombin time; RC, red color sign; RC0, no redness observed at all; RC1, a few localized lesions; RC2, between RC1 and RC3; RC3, multiple lesions observed around the entire circumference; SB‐tube, sengstaken‐blakemore tube; SGV, short gastric vein; T‐bil, total bilirubin.

### Development of varices simple models

A polyethylene resin (thickness, 0.045 mm) was cut into a cylindrical shape, and gel was injected inside to simulate EV. For the purpose of evaluating the effectiveness of blood flow occlusion during EVL based on variceal size, EV simple models with varying variceal diameters (10, 15, 20, and 25 mm) were created, and the red liquid resembling blood was passed through the inside of the EV model. The EVL was performed using the Pneumo‐Activate EVL device (MD‐48709U; SB Kawasumi Corporation). To evaluate the effectiveness of blood flow occlusion during EVL based on the size of the varices, EV simple models with different varicose diameters (10, 15, 20, and 25 mm) were constructed and filled with a red liquid to resemble blood.

### Literature search

Searches were conducted from 1990 to 2024 using the keywords “pipeline esophageal varices” and “giant esophageal varices” in PubMed, and “pipeline esophageal varices” and “giant tree esophageal varices” in Igaku Chuo Zasshi to review the previously reported cases.

## RESULTS

### Characteristics of patients with EVs

Overall, 179 patients had typical EVs, while four had pipeline EVs (palisading vessels were used to define typical EVs; Table 1).

In terms of treatment, 140 patients underwent EIS, 42 received EVL, and one patient was treated with a Sengstaken‐Blakemore (SB) tube. The breakdown of EVL cases was as follows: 31 patients had poor liver function and were not candidates for EIS, 10 had no blood vessels suitable for EIS, and one had portal vein thrombosis (Vp4), precluding the use of EIS. The SB‐tube was used in a case where a CT scan indicated a high risk of EV rupture, but the patient's poor level of consciousness made endoscopic treatment difficult.

The median diameter of the pipeline EVs was larger at 17.5 mm (range, 10–25 mm) compared to the typical varices group, which had a median diameter of 6 mm (range, 3–13 mm). In the typical varices group, the LGV was the only collateral route in 88.3% of cases, while it was the only collateral route in 100% of cases in the pipeline varices group. Additionally, the median diameter of the LGV in the pipeline EVs group was larger at 10 mm (range, 7–20 mm) compared to 5 mm (range, 2–12 mm) in the typical varices group.

The rate of liver cirrhosis, ascites, and hepatocellular carcinoma in the typical varices and pipeline EVs group were 93.3%/75.0%, 33.0%/0%, and 20.7%/25.0%, respectively. The median platelet count (10^3^/µL) was 88 (range, 23–439) in the typical EVs group and lower at 74 (range, 53–102) in the pipeline EVs group.

### Characteristics of patients with pipeline EVs at our hospital

**FIGURE 1:**
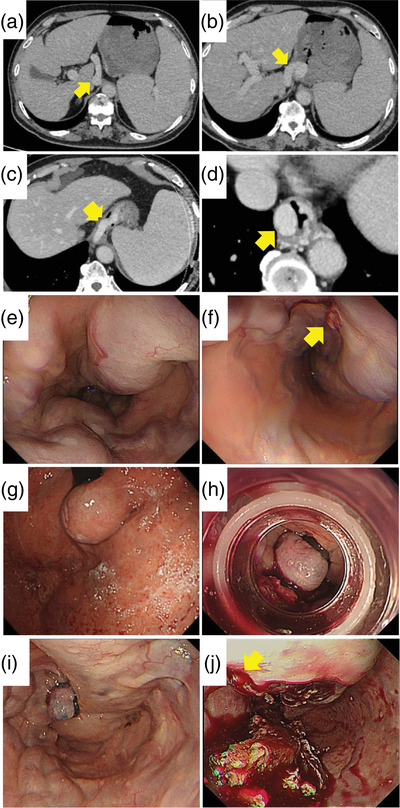
A 70‐year‐old man was admitted with complaints of hematemesis. Pipeline esophageal varix (EV) measuring 25 mm in diameter was identified in contrast‐enhanced computed tomography (a–d). In the emergency upper endoscopy (e–i), hemostasis was achieved at the site of the red plug using endoscopic variceal ligation (EVL). On day 8, endoscopic injection sclerotherapy was performed using the ethanolamine oleate method, but the sclerosing agent failed to stagnate within the blood vessels. Absolute ethanol was intermittently injected in 1 mL increments (a total of 3 mL), but the treatment proved ineffective. EVL was performed at the puncture site, completing the procedure. On day 12, the patient experienced hematemesis, prompting another emergency upper endoscopy. The initial EVL site had ulcerated, and active bleeding was observed nearby (j). EVL was difficult due to the proximity to the ulcer. An attempt to place a Sengstaken‐Blakemore tube was made, but the patient's blood pressure dropped during preparation, and he died. (a) Dilated left gastric vein (arrow). (b) Left gastric vein flowing into the gastric cardia (arrow). (c) Left gastric vein continuing into the esophagus without passing through blind vessels (arrow). (d) Dilated EVs (arrow). (e) Pipeline EV visible at the 2 o'clock position. (f) Red plug on the pipeline EV (arrow). (g) Varices in the gastric cardia. (h) EVL performed on the red plug area. (i) Image showing hemostasis after EVL. (j) Upper endoscopy image during rebleeding, with bleeding observed near the ulcer after EVL (arrow).

Of the four cases, EIS was performed in two (Case 2 and Case 3; Table 2). In the other two cases (Case 1 and Case 4), the sclerosing agent failed to remain in the blood vessels. In Case 1, the procedure was completed by performing EVL at prominent RC sign locations. In Case 4, after EIS treatment, rebleeding occurred near an ulcer after EVL during an emergency endoscopy, and the patient died. Compared with cases in which the sclerosing agent was successfully injected during EIS (Case 2, varices diameter 15 mm; Case 3, varices diameter 10 mm), the diameter of the varices and collateral LGV diameter was larger in cases where EIS was difficult (Case 1, varices diameter 20 mm; Case 4, varices diameter 25 mm). As a representative case of pipeline EVs at our hospital, we described the details of Case 4 in Figure 1.

**TABLE 2:** The cases of pipeline esophageal varices at our hospital.

			Blood biochemistry								Endoscopic treatment alone	
No.	Age/sex	Etiology	T‐bil level (mg/dL)	Alb level (g/dL)	PLT count (10^3^/µL)	PT%	Child‐Pugh grade/mALBI Grade	Morphology	Size of varices (mm)	Blood supply route/size (mm)	Success/failure of EIS	Treatment
1	68/M	IPH	0.8	4.4	99	102	Grade A/ Grade 1	F3LsCbRC2, Lg+	20	LGV/10	Failure	Two courses of the EO method were unsuccessful. EVL was administered for two courses.
2	68/M	HCV	0.9	3.2	86	88	Grade A/ Grade 2b	F3LmCbRC1, Lg+	15	LGV/9	Success	The EO method for two courses and the AS method for one course.
3	57/F	HCV	1.2	3.3	63	74	Grade A/ Grade 2b	F3LmCbRC2, Lg−	10	LGV/7	Success	The EO method for two courses and the AS method for one course.
4	70/M	IPH	1.2	3.6	45	53	Grade A/ Grade 2b	F3LmCbRC1, Lg+	25	LGV/20	Failure	Unsuccessful with ET method + EISL

Abbreviations: Alb, albumin; AS, aethoxysklerol; Cb, blue varices; EIS, endoscopic injection sclerotherapy; EISL, endoscopic injection sclerotherapy with ligation; EO, ethanolamine oleate; ET, absolute ethanol; EVL, endoscopic variceal ligation; F3, large/coil‐shaped; LGV, left gastric vein; mALBI, modified albumin‐bilirubin; PLT, platelet count; PT, prothrombin time; RC1, a few localized lesions; RC2, between RC1 and RC3; T‐bil, total bilirubin.

### Study using an EV simple models

**FIGURE 2:**
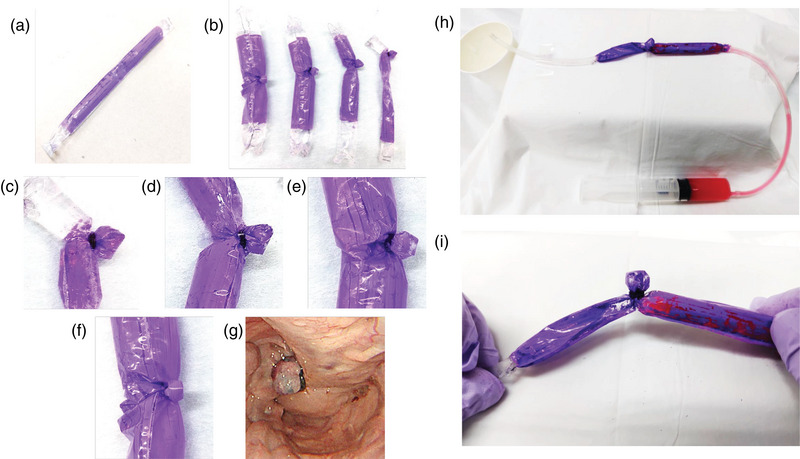
Creation of esophageal varix (EV) simple models. (a) EV model made from polyethylene. (b) Models of EV in various sizes (10, 15, 20, and 25 mm from right to left). (c) Enlarged photograph of a 10 mm diameter EV model. (d) Enlarged photograph of a 15 mm diameter EV model. (e) Enlarged photograph of a 20 mm diameter EV model (f) Enlarged photograph of a 25 mm diameter EV model. (g) EV in Case 4 (maximum diameter, 25 mm). (h) EV model that evaluates the effectiveness of blood flow occlusion by flowing a red liquid resembling blood inside the model. (i) Image of endoscopic variceal ligation in the 15 mm diameter model.

An esophageal varices model was created by forming polyethylene resin into a cylindrical shape and injecting gel inside (Figure 2a). The EVL device had an approximate diameter of 10 mm, and models with varices of varying diameters (10, 15, 20, and 25 mm) were constructed to simulate varices larger than the EVL device (Figure 2b).

For the 10 mm diameter model, the entire varix was successfully drawn into the device and ligated (Figure 2c). In the 15 mm model, most of the varix could be ligated, though some areas remained connected to the gel (Figure 2d). For the 20 mm (Figure 2e) and 25 mm (Figure 2f) diameter models, only portions of the varices were ligated, with the gel remaining connected.

When comparing the ligation image of the 25 mm varix model (Figure 2f) with the endoscopic image of the 25 mm esophageal varix from Case 4 (Figure 2g), the EVL ligation pattern appeared to accurately replicate the ligation observed in the clinical case. The red liquid resembling blood was passed through the inside of the EV model to evaluate the effectiveness of blood flow occlusion during EVL based on variceal size (Figure 2h). Image of EVL ligation in the 15 mm diameter model (Figure 2i).　EVL provides good blocking of the red liquid (Figure 2i).

### Literature search

Literature searches identified 10 peer‐reviewed cases of pipeline EVs. These reports, along with our cases, were included in the analysis, resulting in a total of 14 cases (Table 3).

**TABLE 3:** Literature review of the cases of pipeline esophageal varices.

No.	Year	Authors	Age/sex	Etiology	Morphology	Size of varices (mm)	Blood supply route/size (mm)	Endoscopic treatment alone		IVR + ecndoscopic or surgical treatment
								Success/failure of EIS	Treatment	
	
1	1991	Shimakawa et al.^11)^	41/M	Alcohol	F3LsCbRC1, Lg−	–	LGV/–	Failure	Five courses of the EO method were unsuccessful.	PSE: Transabdominal esophageal transection
2	1996	Hamamoto et al.^14)^	67/F	HBV	F2LsCbRC1, Lg+	–	LGV/–	Success	Eight courses of the EO method were successful.	
3	1997	Umezawa et al.^15)^	42/M	HCV	F2LsCbRC2, Lg+	–	LGV, SGV/–	Failure	Nine courses of the EO method were unsuccessful.	TIPS: Success with the EO method
4	1997	Umezawa et al.^15)^	63/F	HCV	F3LsCbRC2, Lg+	–	LGV/–	No endoscopic treatment		TIPS: Success in 5 courses of EO method
5	1999	Hamaguchi et al.^4)^	74/F	HBV	F2LmCbRC1, Lg+	8	LGV/–	Success	One course of EISL was successful.	
6	2004	Suda et al.^16)^	81/M	–	F3LmCbRC1, Lg−	–	–	Success	Three courses of the ETP method were successful.	
7	2005	Shudo et al.^17)^	60s/M	Alcohol	F2LmCbRC2, Lg−	–	LGV/–	No	Two courses of the selective EVL method were successful.	
8	2006	Imai et al.^6)^	64/F	HCV	F3, Lg+	–	LGV/–	Success	Three courses of the EO method were unsuccessful. Success with N‐butyl‐2‐cyanoacrylate injection + EO method.	
9	2022	Yokoyama et al.^18)^	29/M	EPVO	F3LsCbRC3, Lg+	–	LGV/–	Failure	One course of the EO method was successful.	Success with the TIO + EO method
10	2023	Chikamori et al.^19)^	53/M	Alcohol+ HCV	F3CbRC1, Lg+	–	LGV/–	No endoscopic treatment		Success with the PSE + EO method
11	2024	Our case (Case 1)	68/M	IPH	F3LsCbRC2, Lg+	20	LGV/10	Failure	Two courses of the EO method were unsuccessful. EVL was administered for two courses.	
12	2024	Our case (Case 2)	68/M	HCV	F3LmCbRC1, Lg+	15	LGV/9	Success	The EO method for two courses and the AS method for one course.	
13	2024	Our case (Case 3)	57/F	HCV	F3LmCbRC2, Lg−	10	LGV/7	Success	The EO method for two courses and the AS method for one course.	
14	2024	Our case (Case 4)	70/M	IPH	F3LmCbRC1, Lg+	25	LGV/20	Failure	Unsuccessful with ET method + EISL	

Abbreviations: AS, aethoxysklerol; EISL, endoscopic injection sclerotherapy with ligation; EO, ethanolamine oleate; ET, absolute ethanol; ETP, ethanol‐thrombin‐polydocanol; IVR, interventional radiology; LGV, left gastric vein; PSE, partial splenic embolization; SGV, short gastric vein; TIO, transileocolic obliteration; TIPS, transjugular intrahepatic portosystemic shunt.

## DISCUSSION

Specific treatment guidelines for pipeline EVs have not yet been established. In this study, we reviewed both cases of pipeline EVs treated at our hospital and previously reported cases. Our findings indicated that EIS was challenging in many cases of pipeline EVs, and many required a combination of IVR with either endoscopic or surgical treatment. Additionally, we developed EV simple models to investigate the efficacy of EVL in achieving sufficient ligation and blood flow occlusion in large EVs, such as pipeline EVs. Our study using EV simple models suggested that EVL was highly effective in blocking blood flow to EVs with a diameter of 15 mm or less. When performing endoscopic treatment of EVs, it is important to understand that there are limitations to occluding blood flow by EVL in some EVs.

Among the 183 cases of EVs treated at our hospital, 4 (2.2%) were identified as pipeline EVs, consistent with previously reported proportions. All four cases were classified as Child‐Pugh grade A, indicating preserved liver function. However, the platelet count (10^3^/µL) was notably decreased, with a median of 74. This suggests that, in addition to preserved hepatic reserve, severe portal hypertension may contribute to the development of pipeline EVs.

In cases where EIS was difficult, both the EVs and collateral passage diameters were wide. We reconsidered whether a treatment strategy centered around EIS, as used for typical EVs, is appropriate for managing pipeline EVs. We investigated previously reported treatment methods for pipeline EVs.

According to the literature search, 8 out of 14 cases (57.1%) were treated with endoscopic intervention alone. Six of these eight cases were primarily treated with EIS, including one case of endoscopic injection sclerotherapy with ligation (EISL), two cases using the EO method, one case using the ethanol–thrombin–polydocanol method, one case using the selective EVL method, and one case involving *N*‐butyl‐2‐cyanoacrylate injection.

In six cases (42.9%), endoscopic treatment alone was insufficient to complete the treatment. Of these, five cases (35.7%) required additional interventions, including four cases where endoscopic treatment was performed following interventional radiology, and one case that required surgical treatment after interventional radiology. These findings suggest that endoscopic treatment alone may be inadequate in certain cases of pipeline EVs, highlighting the need for combined therapeutic approaches.

Since pipeline EVs have rich blood flow and are at high risk of bleeding from the puncture site, EISL is recommended.^5^ Additionally, Obara et al. reported that before sclerotherapy, the presence of an external esophageal shunt should be identified using endoscopic ultrasonography. If the perforating vessel measures 3–5 mm, the selective EVL method, which involves anhydrous ethanol injection (ET method) to ligate the perforating vessel, is effective. For vessels 5 mm or larger, the combined selective EVL and EO method (selective EVL/EO combined method) is recommended.^1^ There have also been reports of successful treatment using the EO method after N‐butyl‐2‐cyanoacrylate injection, especially in cases where the variceal protuberance is pronounced, and blocking the perforating vessels using the selective EVL method is difficult.^6^

EVs are primarily treated with endoscopic therapy; however, in cases where this approach is difficult, IVR or surgical treatment may be necessary. Partial splenic embolization is used for both EVs and GVs, effectively reducing blood flow in the splenic vein and lowering portal vein pressure.^8^ For refractory EVs and GVs, embolization of the blood supply route is performed using percutaneous transhepatic obliteration or transileocolic obliteration. However, the frequency of these procedures has decreased due to their high invasiveness.^9^ Transjugular intrahepatic portosystemic shunt is considered effective for refractory EVs,^10^ but the number of cases performed in Japan remains low.　Surgical treatments, including esophageal transection and the Hassab procedure,^11^ have also declined in prevalence due to the spread of IVR, which is less invasive.^8^ There are various approaches to treating refractory EVs, including interventional radiology and surgery, but the current situation is that treatment policies vary depending on the medical system of each facility.

In a review summarizing treatment cases of pipeline EVs at our hospital and previously reported treatment cases of pipeline EVs at other facilities, treatment with endoscopy alone was difficult in 42.9% of cases, and when endoscopic treatment was difficult for pipeline EVs, treatment including IVR and surgery was performed. In our cases, the varices diameter was large in all of the cases in which endoscopic sclerotherapy was unsuccessful (Case 1: 20 mm, Case 4: 25 mm), and we thought that the size of the varices diameter was one of the factors that made endoscopic treatment difficult. However, there were few reports that specified the size of the varices diameter, making it difficult to consider the thought. At present, in our review of treated cases of pipeline EVs, it is not possible to compare successful cases of endoscopic treatment alone with unsuccessful cases, and it has not yet been possible to clarify the treatment selection criteria for pipeline EVs.

**FIGURE 3:**
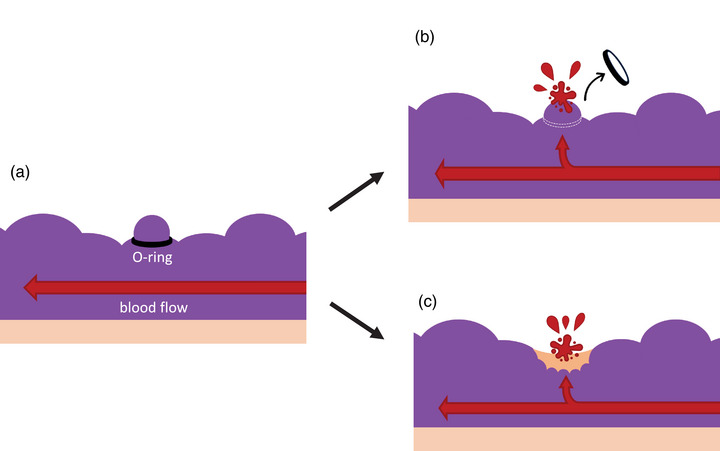
Illustrations of unsuccessful cases of ligation and blood flow occlusion. (a) Poor ligation and blood flow occlusion using endoscoic variceal ligation (EVL). The varices diameter is larger than that of the EVL band (O‐ring), leading to inadequate ligation. (b) The EVL band may fall off. (c) EVL does not completely block blood flow with a high risk of rebleeding from the ulcer.

Pipeline EVs have a large blood flow rate and high velocity, leading to significant bleeding from the puncture site during EIS, necessitating EVL to control the bleeding. However, we recently encountered a case (Case 4) where a patient died due to rebleeding near an ulcer after EVL, raising concerns about the safety of performing EVL on large‐diameter EVs. If the entire varix cannot be retracted into the device during EVL, or if blood flow cannot be blocked, there is a risk of the EVL band detaching or subsequent bleeding from the ulcer (Figure 3). GVs are typically larger in size than EVs, making it difficult to retract them fully into the device. Consequently, ulcers often form at the EVL site, leading to a recommendation against EVL for GVs.^7^ While EVL is a well‐established method for controlling hemorrhage in EVs, there may be specific cases in which it is not advisable for certain types of EVs, similar to GVs.

We created the EV simple models and investigated whether EVL could be used to sufficiently ligate and occlude blood flow in large EVs such as pipeline EVs. We confirmed that EVL was able to block the liquid flow in the EV models with a diameter of 15 mm or less. On the other hand, we observed that performing EVL on EV with a diameter of 20 mm or more leads to insufficient occlusion of liquid flow, thereby increasing the risk of post‐EVL complications (such as rebleeding from EVL band dislodgment or ulcers). This study suggested that EVL is effective in blocking blood flow for EVs with a diameter of 15 mm or less. However, this is only a study using EV simple models, and it is desirable to accumulate clinical data evaluating the diameter of EVs and the degree of ligation by EVL.

In Case 4, rebleeding from the ulcer margin after EVL complicated further EVL attempts, raising questions about what alternative measures could have been taken to save the patient's life.

Notably, EVL is not feasible in all cases; if the bleeding point is not completely addressed, the situation may worsen, and the applied band may prevent a second endoscopic intervention. N‐butyl‐2‐cyanoacrylate has been approved in many countries as a sclerotherapy for esophageal and gastric (fundal) varices, although it has yet to receive approval for EVs in Japan. Although there are reports of the efficacy of *N*‐butyl‐2‐cyanoacrylate for EVs in Japan,^6^ few facilities currently utilize it. When pipeline EVs rupture, *N*‐butyl‐2‐cyanoacrylate injection serves as a salvage therapy for immediate bleeding control or as a reserved treatment for high‐risk patients in whom endoscopic treatment is difficult.^12^, ^13^ Therefore, it is essential to obtain sufficient consent from the patient and their family in such situations.

This study has some limitations. First, the number of cases of pipeline EVs at our hospital and other facilities reported in previous reports is small, and we were unable to identify the characteristics of cases that are difficult to treat with endoscopy alone, so we were unable to determine the treatment strategies for pipeline EVs. We thought that further accumulation of cases would be necessary in the future. Second, the EV model created in this study is very simple and the model does not reproduce the properties of EVs, and this model was created for the purpose of determining the safe ligation diameter for EVL. In the future, it will be necessary to create a model that more faithfully reproduces the properties of EVs, and it is desirable to accumulate data evaluating the diameter of EVs and the degree of ligation by EVL.

Pipeline EVs may be difficult to treat with endoscopic therapy alone, and IVR or surgical intervention may be necessary in some cases. There are few reported cases of pipeline EVs, making it difficult to determine the treatment strategies. When performing endoscopic treatment, if the EV diameter is large, the risk of post‐EVL complications is expected to be high. In our study using EV simple models, it was suggested that EVL is effective in blocking blood flow for EVs with a diameter of 15 mm or less. It is significant that we understand there are EVs for which there are limitations to occluding blood flow with EVL, and it may be necessary to consider strategies that include treatment methods other than endoscopic therapy. Further accumulation of treatment data for pipeline EVs is necessary to refine management strategies.　The results of our study are expected to guide the treatment plan for pipeline EVs.

## CONFLICT OF INTEREST STATEMENT

None.

## ETHICS STATEMENT

This retrospective study was approved by the Ethics Review Committee of Yamagata University School of Medicine (approval number: 2019–316).

## PATIENT CONSENT STATEMENT

All patients provided informed consent for the use of their clinical information for research purposes.
